# Acute symptomatic hyponatremia in setting of SIADH as an isolated presentation of COVID-19

**DOI:** 10.1016/j.idcr.2020.e00859

**Published:** 2020-06-01

**Authors:** Mhd Baraa Habib, Sundus Sardar, Jamal Sajid

**Affiliations:** a Internal Medicine Residency Program, Internal Medicine Department, Hamad Medical Corporation, Doha, Qatar; b Internal Medicine Department, Hamad Medical Corporation, Doha, Qatar

**Keywords:** COVID-19, Coronavirus disease 2019, SARS-CoV-2, severe acute respiratory syndrome coronavirus 2, SIADH, syndrome of inappropriate antidiuretic hormone secretion, ECG, electrocardiogram, Hyponatremia, SIADH, COVID-19, Novel coronavirus, SARS-CoV-2 infection, Atypical presentation

## Abstract

The syndrome of inappropriate secretion of antidiuretic hormone (SIADH) is one of the most common causes of hyponatremia in hospitalized patients. Wide spectrum of etiologies associated with hyponatremia pose significant challenges in detecting and treating this disorder. Several infectious causes of SIADH have been reported; however, hyponatremia associated with SIADH and Coronavirus disease 2019 (COVID-19) was only recently mentioned in a few case reports. We discuss a unique presentation of COVID-19, in which the patient presented with acute severe symptomatic hyponatremia thought to be the initial and isolated presentation of SARS-CoV-2 infection.

## Introduction

The syndrome of inappropriate secretion of antidiuretic hormone (SIADH) is characterized by euvolemic hyponatremia, low plasma osmolality, high urinary osmolality, elevated natriuresis, hypouricemia, and lack of evidence of other hyponatremic diseases. This syndrome occurs in response to continued antidiuretic hormone (ADH) release in spite of low serum osmolality. Central nervous system disorders, pneumonia, endocrine diseases, paraneoplastic syndromes, and various drugs may cause SIADH [1].

Coronavirus disease 2019 (COVID-19), which is caused by severe acute respiratory syndrome coronavirus 2 (SARS-CoV-2), transmitted often from human to human by droplet and contact routes [2]. Currently, RNA detection by highly sensitive RT-PCR, has been pivotal for the diagnosis of COVID-19. This disease usually manifests as upper or lower respiratory system illness in majority of clinical presentations [3]. The most common symptoms associated with COVID-19 infection are fever, congested nose, and cough; and may also cause severe pneumonia [4]. We describe the case of a middle-aged gentleman with acute symptomatic hyponatremia and SIADH, attributed to COVID-19.

## Case report

A 57-year-old man, with past medical history significant for hypertension on amlodipine and type II diabetes on insulin glargine, presented to the emergency department with one day history of acute dizziness, most prominent with head movements and exacerbated with standing. It was associated with retrosternal burning pain especially after meals. At that time, he was investigated for coronary artery disease, with normal ECG and negative serial troponins. Serum sodium was 128mEq/L (reference range 135–145 mEq/L), however, it was not investigated initially. He was discharged on betahistine.

Three days later, he presented again with increasing severity of dizziness, along with nausea, fatigue, and generalized headache. There was no fever, chills, cough or shortness of breath. The patient denied any change of his oral intake of fluid, introduction of new medications or change in his urinary habits. He was afebrile, with blood pressure of 152/90 mmHg, heart rate of 88 beats per minute and respiratory rate of 19 breaths per minute with oxygen saturation of 98 % on room air. Physical exam was unremarkable, with no signs of overload or dehydration.

Laboratory findings were significant for serum sodium of 112 mEq/L (normal range 136–145 mEq/L). Three days before his serum sodium was 128 mEq/L, and 137 mEq/L noted on a prior admission for a urinary tract infection three years ago. Laboratory investigations revealed normal BUN of 8.96 mg/dL (reference range: 7–20 mg/dL) and creatinine of 0.79 mg/dL (normal range: 0.5–1.2 mg/dL). Random blood glucose was 9.6 mmol/L. Further workup revealed urine osmolarity of 237 mOsm/kg, urinary sodium 63 mEq/L with low serum osmolality of 240 mOsm/kg. Thyroid stimulating hormone and ACTH stimulation test were normal. A chest X-ray did not show any infiltrates. Electrocardiogram showed no abnormality. Head computed tomography scan was unremarkable. As COVID-19 was widespread in the community of Qatar, and a case series in our center recently reported an association between COVID-19 and SIADH, we sent a nasopharyngeal swab for SARS-CoV-2 PCR which came back positive. Since all workup for pulmonary and central nervous system disorders remained negative, the patient was diagnosed as having SIADH due to underlying COVID-19 infection.

The treatment with slow hypertonic saline 3% infusion was initiated with remarkable improvement in his symptoms. Oral fluid restriction was initiated. Serum sodium levels were monitored for the next 72 h, and fluctuated between 124–130 mEq/L. The patient was transferred to a quarantine facility; however, he did not develop any respiratory symptoms or fever.

## Discussion

Multiple etiologies for hyponatremia in SIADH patients have been suggested such as malignancy, pulmonary conditions, central nervous system disorders, and medications [1]. Diagnostic criteria for SIADH comprises of laboratory tests such as serum sodium level, serum osmolality, urine sodium level, urine osmolality, serum cortisol and thyroid function tests, in addition to a detailed history, physical exam for volume status and medications [1].

Our patient was diagnosed as SIADH based on hyponatremia (serum sodium level <135 mmol/l), serum osmolality < 275 mOsm/kg, urine osmolality >100 mOsm/kg, urine sodium concentration >40 mEq/l, and euvolemic state with normal renal, adrenal, and thyroid functions. Although many medications may cause hyponatremia, all medications our patient was taking are not implicated to induce hyponatremia, and the patient was compliant to the same medications for a long time without any recent changes.

It is well known that COVID-19 presentation can vary from asymptomatic to severe pneumonia [5]. A meta-analysis showed that the main clinical symptoms of COVID‐19 patients were fever (88.5 %), cough (68.6 %), myalgia or fatigue (35.8 %), expectoration (28.2 %), and dyspnea (21.9 %). Minor symptoms include headache or dizziness (12.1 %), diarrhea (4.8 %), nausea and vomiting (3.9 %) [6]. Hyponatremia is frequently associated with atypical pneumonia. One of the underlying mechanisms is inappropriate ADH secretion [7].

A case series of COVID-19 pneumonia associated with syndrome of inappropriate antidiuretic hormone secretion (SIADH) published recently described patients who presented with respiratory symptoms, were diagnosed with COVID-19, and found to have SIADH [8]. However, in all mentioned cases, fever was a common feature and there was evidence of pneumonia (abnormal chest X-ray depicting bilateral infiltrates) with no symptoms pertaining to hyponatremia. In our case, the patient presented to the hospital with complaint of symptoms related to the underlying hyponatremia, with no fever, cough or shortness of breath. He was found to have acute severe hyponatremia of SIADH. After excluding other causes of SIADH in our case, we suggest that SARS-CoV-2 infection is the cause of SIADH with this presentation of acute severe hyponatremia.

Although the relation between COVID-19 and SIADH was mentioned in a few cases [8], the unique presentation of COVID with only symptoms of hyponatremia and negative chest x-ray has not been reported previously. We conclude that COVID-19 can present with symptomatic hyponatremia even without other classical symptoms and signs of the disease.

## Conclusion

SIADH is an important complication of COVID-19 and could be the first and only manifestation. The presence of SIADH could be a clue for diagnosing COVID-19. In cases of SIADH with no clear etiology, it is reasonable that physicians maintain a low threshold to test for COVID-19 as a possible cause of SIADH.

Association between COVID-19 and SIADH needs to be further identified, requiring clinicians to be aware of this condition. Additional studies are required to ascertain the incidence and pathogenesis of SIADH in patients with COVID-19 so that specific treatment protocols may be devised for management of hyponatremia in these cases.

## Authorship contributions

Writing the initial draft of the manuscript: Mhd Baraa Habib, Sundus Sardar

Conceptualization and supervision: Jamal Sajid

Medical management of the case: Jamal Sajid, Mhd Baraa Habib

Revising the manuscript critically and literature review: Sundus Sardar, Mhd Baraa Habib, Jamal Sajid

The first (MBH), corresponding (SS), and the senior author (JS) contributed equally to the writing and preparation of this article. MBH and SS have written the initial draft of the manuscript and attempted the literature review. The draft was revised and updated by JS. MBH and JS were part of the medical treating team. All the authors critically reviewed the initial and the final draft of the manuscript and approved it for submission.

## Consent

Written informed consent was obtained from the patient for publication of this case report. A copy of the written consent is available for review by the Editor-in-Chief of this journal.

## Declaration of Competing Interest

The authors have no conflict of interest relevant to this case.

## Funding

Qatar National library funded the open access publication fees of this case.
